# Custom-Made 3D-Printed Implants for Paprosky Type 3A and 3B Acetabular Defects: Early Surgical and Functional Outcomes from a Multicentre Study

**DOI:** 10.3390/medicina62091723

**Published:** 2026-09-07

**Authors:** Grzegorz Guzik, Piotr Szremski, Daniel Pyrka, Paweł Łęgosz

**Affiliations:** 1 Department of Orthopaedic Oncology, Subcarpatian Oncology Centre, 36-200 Brzozow, Poland; 2 Department of Orthopaedic Oncology, Specialist Hospital in Brzozów- Podkarpacie Oncology Centre, Dworska 77A, 38-420 Korczyna, Poland; 3 Trauma and Orthopaedic Department, District Hospital in Przemyśl, 37-700 Przemyśl, Poland; pszremski@wszp.pl; 4 Trauma and Orthopaedic Department, District Hospital in Krosno, 38-400 Krosno, Poland; drdanielpyrka@gmail.com; 5 Orthopedics and Traumatology Clinic in Warsaw, Medical University of Warsaw, 02-091 Warsaw, Poland; ortopedia.skdj@uckwum.pl

**Keywords:** acetabular bone defect, revision total hip arthroplasty, custom-made implant, 3D printing, Paprosky classification, osseointegration

## Abstract

*Background and Objectives:* Reconstruction of extensive acetabular bone defects in revision total hip arthroplasty (THA) remains technically demanding and is associated with a high risk of complications. Custom-made three-dimensional (3D)-printed implants have emerged as a patient-specific option for managing severe bone loss, particularly in Paprosky type 3A and 3B defects. The aim of this multicentre study was to evaluate the early clinical, radiographic, and surgical outcomes following single-stage reconstruction using custom-made 3D-printed acetabular implants. *Materials and Methods:* This retrospective multicentre study included 62 patients treated between 2021 and 2024 at four orthopaedic departments. All patients had Paprosky type 3A (*n* = 34) or 3B (*n* = 28) acetabular defects and underwent reconstruction with custom-made 3D-printed implants. A minimum clinical follow-up of 6 months was required for inclusion. Preoperative computed tomography was used for implant design and surgical planning. Clinical assessment included pain using the Visual Analogue Scale, functional evaluation using the Harris Hip Score and Karnofsky Performance Scale, mobility, range of motion, and use of orthopaedic aids. Radiographic evaluation included plain radiographs and computed tomography. Standardised functional and CT outcome assessments were performed during the first 6 months after surgery, whereas the mean overall follow-up represented subsequent clinical surveillance. Mean follow-up was 27 months. *Results:* All patients had undergone multiple previous surgical procedures before implantation of the custom-made prosthesis. The interval between primary arthroplasty and implantation of the custom-made implant ranged from 9 to 31 years. During follow-up, improvements in pain, mobility, and functional performance were observed across the entire cohort. The reported functional and CT outcomes refer to the standardised 6-month assessment period. Radiographic evaluation demonstrated satisfactory implant positioning and restoration of hip biomechanics. Progressive radiographic findings suggestive of implant integration were observed during follow-up. The most common complications were postoperative wound-healing disorders and prosthetic dislocation, particularly in patients with more extensive bone defects. *Conclusions:* Single-stage reconstruction using custom-made 3D-printed implants appears to be a feasible treatment option for patients with Paprosky type 3A and 3B defects, resulting in improvements in pain, mobility, and functional performance. Radiographic assessment suggested stable implant fixation and progressive implant integration during early follow-up. However, postoperative wound-healing disorders, infection, and prosthetic dislocation remain clinically relevant complications, particularly in patients with more extensive bone defects. Further studies with longer follow-up are required to determine long-term implant durability, implant survivorship, and functional outcomes.

## 1. Introduction

Aseptic loosening of hip prostheses is one of the most common indications for revision surgery after total hip arthroplasty (THA) [1]. Extensive acetabular bone defects may result from multiple previous revision procedures, severe osteolysis, pelvic trauma, or tumour resection [2,3]. Because revision procedures are associated with a high risk of complications, meticulous preoperative planning and the use of advanced surgical techniques are essential to optimise outcomes [4].

The extent of acetabular bone loss is most commonly assessed using the Paprosky classification [5]. This system is based on preoperative radiographs and categorises defects according to the degree of acetabular component migration and the integrity of key supporting structures, including the anterior and posterior columns, the superior weight-bearing dome, and the medial wall [6,7].

Management of acetabular bone defects typically involves the use of bone grafts and bone substitutes; however, graft incorporation is often slow and may be incomplete [8,9]. Consequently, revision systems have received increasing attention in recent years. These systems generally include hemispherical acetabular components fixed with screws, anti-protrusio cages, augments, and modular revision implants [10,11,12,13,14,15]. Despite ongoing improvements in implant design, reconstruction of Paprosky type 3A and 3B defects remains associated with substantial technical difficulty and variable clinical outcomes. Reported survival rates for these implants range from 50% to 98% [15].

Custom-made and non-standard implants manufactured using three-dimensional (3D) technologies have been used successfully since the 1990s [16,17]. Second-generation designs have demonstrated improved radiological and functional outcomes [18,19]. The advantages of these implants include the possibility of single-stage reconstruction of acetabular defects, restoration of pelvic stability, achievement of stable primary fixation using screws and pins, and reconstruction of the native acetabular geometry. In addition, advances in biomaterials have led to the development of porous implant surfaces that mimic cancellous bone, thereby enhancing osseointegration [19,20].

Although the use of custom-made 3D-printed implants has increased substantially in recent years, evidence regarding their early clinical and radiographic performance in patients with severe Paprosky type 3A and 3B defects remains limited. In particular, multicentre data directly comparing early functional, radiographic, perioperative, and complication outcomes between Paprosky type 3A and 3B defects remain scarce.

The aim of the present study was to evaluate the efficacy of single-stage reconstruction using custom-made 3D-printed implants in patients with extensive acetabular bone defects classified as Paprosky type 3A or 3B. Functional, radiographic, and surgical outcomes were assessed, and perioperative complications were analysed. We hypothesised that the use of custom-made 3D-printed implants would result in favourable functional outcomes and acceptable complication rates.

## 2. Material and Methods

Between 2021 and 2024, 69 hip reconstruction procedures using custom-made three-dimensional (3D)-printed implants were performed at four orthopaedic departments specialising in the treatment of extensive acetabular bone defects. All procedures were performed by surgeons with extensive experience in this field.

This study was conducted as a retrospective analysis of anonymized patient data. In accordance with national regulations, formal ethics committee approval was not required. All patients provided written informed consent to participate in the study. The study was designed and reported in accordance with the STROBE (Strengthening the Reporting of Observational Studies in Epidemiology) recommendations for observational studies.The inclusion criteria were extensive acetabular bone defects that were considered difficult to reconstruct using standard implants, Paprosky type 3A or 3B defects, and patients with satisfactory local soft tissue conditions at the surgical site. A minimum clinical follow-up of 6 months was required for inclusion. Despite extensive acetabular bone loss, pelvic discontinuity was not identified in any patient included in the study.

The exclusion criteria included eligibility for alternative surgical procedures, patients in poor general condition who were not suitable for major surgery, septic loosening, active bone infection, active fistula, and patients with elevated inflammatory markers.

Routine preoperative laboratory screening for infection included C-reactive protein (CRP), erythrocyte sedimentation rate (ESR), and white blood cell count (WBC). In cases with equivocal clinical or laboratory findings, leukocyte-labelled scintigraphy was additionally performed to further assess the possibility of occult periprosthetic joint infection. Patients with findings suggestive of active infection were excluded from the study. Routine preoperative microbiological cultures or biopsies were not obtained. Because no single diagnostic test can exclude occult or low-grade periprosthetic joint infection with absolute certainty, infection was assessed using the combined clinical, laboratory, and imaging findings.

All patients had undergone multiple previous procedures because of prosthetic loosening and mechanical implant failure. The number of operations performed before implantation of the custom-made 3D prosthesis ranged from three to eight, with a mean of four procedures. The interval between implantation of the primary prosthesis and implantation of the custom-made implant ranged from 9 to 31 years, with a mean of 16 years. Seven patients had acetabular fractures, and four were diagnosed with pelvic pseudotumours associated with metallosis. Previous surgical approaches included posterior, anterior, combined anterior and posterior, and other approaches. In four historical cases, the available medical documentation described the surgical approach only as “other” and did not allow reliable further classification.

Before surgery, all patients were ambulatory with assistive devices: 54 patients used two crutches, 4 used one crutch, and 4 used a walking frame. A limp was present in all 62 patients and was accompanied by gluteal muscle weakness, as demonstrated by positive Trendelenburg and Duchenne signs. Only seven patients were able to actively raise the leg straight in the supine position. No neurological or vascular deficits were identified. Marked atrophy of the gluteal and thigh muscles was observed in 43 patients.

Preoperative computed tomography (CT) was performed to assess the extent of bone loss. CT examinations were performed using a slice thickness of 1 mm and included the entire pelvis from the iliac crests to the proximal one-third of both femur. The implants were designed collaboratively by medical engineers and orthopaedic surgeons, with particular attention to the planned surgical approach and fixation strategy. The custom-made implants used in the study were manufactured by LimaCorporate S.p.A. (Villanova di San Daniele del Friuli, Udine, Italy) and implantcast GmbH (Buxtehude, Germany). Lima Corporate ProMade implants incorporated Trabecular Titanium technology, whereas Implantcast implants incorporated the porous titanium EPORE structure. These porous surfaces were used in all implants included in the present series to promote bone ingrowth and implant integration. Dual-mobility articulation was used in all reconstructed hips. Primary fixation of the custom-made implants was achieved using screws and/or fixation pins positioned according to the individual preoperative plan and the available bone stock. The participating centres were selected because they used a consistent patient-specific reconstruction strategy and comparable custom-made implant systems. The implant design was approved by the surgical team within approximately 2 weeks, after which manufacturing was initiated. Production required an additional 5 to 6 weeks, after which the implants were available for implantation. No intraoperative navigation system was used.

All index custom-made reconstructions were performed with the patient in the lateral decubitus position through a posterolateral approach. Intraoperative fluoroscopy was not used. Pulsed lavage was not performed.

Antibiotic prophylaxis and thromboprophylaxis were administered in all cases. In addition, all patients received a single preoperative dose of 1 g tranexamic acid. Within the first 24 h after surgery, follow-up radiographs were obtained, and patients were assisted with sitting and early mobilisation. Multimodal analgesia was administered during the first 24 h using an infusion pump delivering oxycodone and morphine. Thereafter, pain management was continued with non-steroidal anti-inflammatory drugs (NSAIDs), metamizole, and tramadol.

A minimum follow-up of 6 months was required for inclusion. Standardised functional and CT outcome assessments were performed during the first 6 months after surgery, whereas the longer follow-up period represented continued clinical surveillance for complications and implant status. Patients were assessed with regard to general condition, pain intensity using the Visual Analogue Scale (VAS), performance status using the Karnofsky Performance Scale (KPS), Harris Hip Score (HHS), mobility, joint range of motion, and use of orthopaedic aids.

A comprehensive analysis of perioperative parameters was performed. This included surgical duration, surgical approach, intraoperative blood loss, blood transfusion requirements, use of surgical drains, perioperative antibiotic prophylaxis, thromboprophylaxis, tranexamic acid administration, and anaesthetic technique.

Pain severity was assessed using the VAS on postoperative day 7 and at 6 weeks, 3 months, and 6 months. Early rehabilitation and ambulation, as well as the need for orthopaedic support, were also evaluated. Passive and active joint range of motion were assessed, with particular attention to the function of individual muscle groups. Functional status was additionally evaluated using the KPS and HHS at the same time points. Postoperative radiographic assessment included plain radiographs obtained on postoperative day 1 and at 6 weeks, 3 months, and 6 months. CT was performed at 3 and 6 months. The accuracy of implant positioning relative to the preoperative plan, implant stability, and implant- or bone-related complications was evaluated.

The centre of rotation (COR) was assessed relative to the planned position, and deviations were measured in millimetres in both the vertical and horizontal planes. Acetabular and femoral offsets were measured relative to the contralateral hip and expressed in millimetres. Acetabular inclination and anteversion were reported as angular values, with anteversion measured using Liaw’s method. Limb length discrepancy was determined relative to the contralateral side and expressed in millimetres as the distance between the tip of the lesser trochanter and the ischial tuberosity.

The following radiographic findings were considered suggestive of implant osseointegration: absence of radiolucent lines greater than 1 mm around the implant and fixation components, absence of implant subsidence greater than 5 mm, absence of implant displacement, and presence of radial trabecular structures on CT.

The DeLee and Charnley classification of periacetabular bone zones was not applied because of the variability in implant geometry. In addition, the use of supplementary fixation elements alters the conditions for implant integration, making this classification unsuitable for comparison with standard cementless acetabular cups.

Surgical complications were recorded and analysed, with particular emphasis on prosthetic dislocation, postoperative wound-healing disorders, infection, bone and implant failure, and injury to pelvic organs, vessels, and nerves.

Technical abbreviations were defined upon first use. A non-blinded assessment of outcomes was performed. Continuous variables were assessed for normality using the Shapiro–Wilk test and are presented as mean ± standard deviation or median with minimal and maximal value as appropriate. Between-group comparisons were conducted using independent-samples *t*-tests or Mann–Whitney U tests for continuous variables and chi-square or Fisher’s exact tests for categorical variables. Repeated measures were analysed using paired *t*-tests or Wilcoxon signed-rank tests. All tests were two-tailed, and *p* < 0.05 was considered statistically significant. All statistical analyses were performed using Statistica version 13 (TIBCO Software Inc. Palo Alto, CA, USA).

## 3. Results

Of the 69 custom-made hip reconstruction procedures initially identified, 62 patients were included in the final analysis. Seven patients were excluded because follow-up contact could not be established. The final cohort comprised 34 patients with Paprosky type 3A defects and 28 patients with Paprosky type 3B defects. The overall clinical follow-up ranged from 7 to 49 months, with a mean of 27 months. Standardised functional and CT outcome assessments were performed during the first 6 months after surgery, whereas the longer follow-up period represented subsequent clinical surveillance for complications and implant status. The study population comprised 38 women and 24 men. The mean age was 76 years in women and 74 years in men (Table 1).

Baseline preoperative assessment demonstrated substantial pain and functional impairment in both groups. Detailed preoperative VAS, HHS, KPS, ambulatory status, limb shortening, and gluteal muscle function are summarised in Table 2.

**Figure 1:**
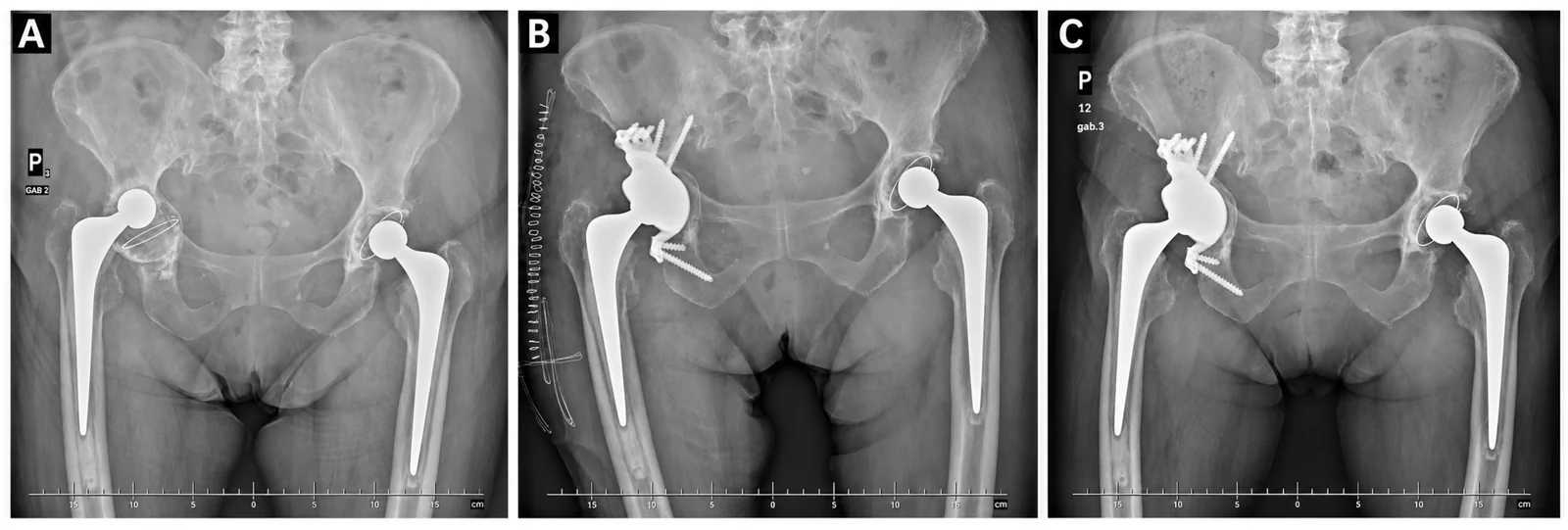
Representative case of a Paprosky type 3A acetabular defect treated with a custom-made 3D-printed acetabular implant. (**A**) Preoperative radiograph demonstrating prosthesis luxation with superior stem migration and extensive acetabular bone loss. (**B**) First day after surgery radiograph—3D custom-made reconstruction and (**C**) one year after surgery demonstrating stable implant position and no sign of implant subsidence or loosening.

**Figure 2:**
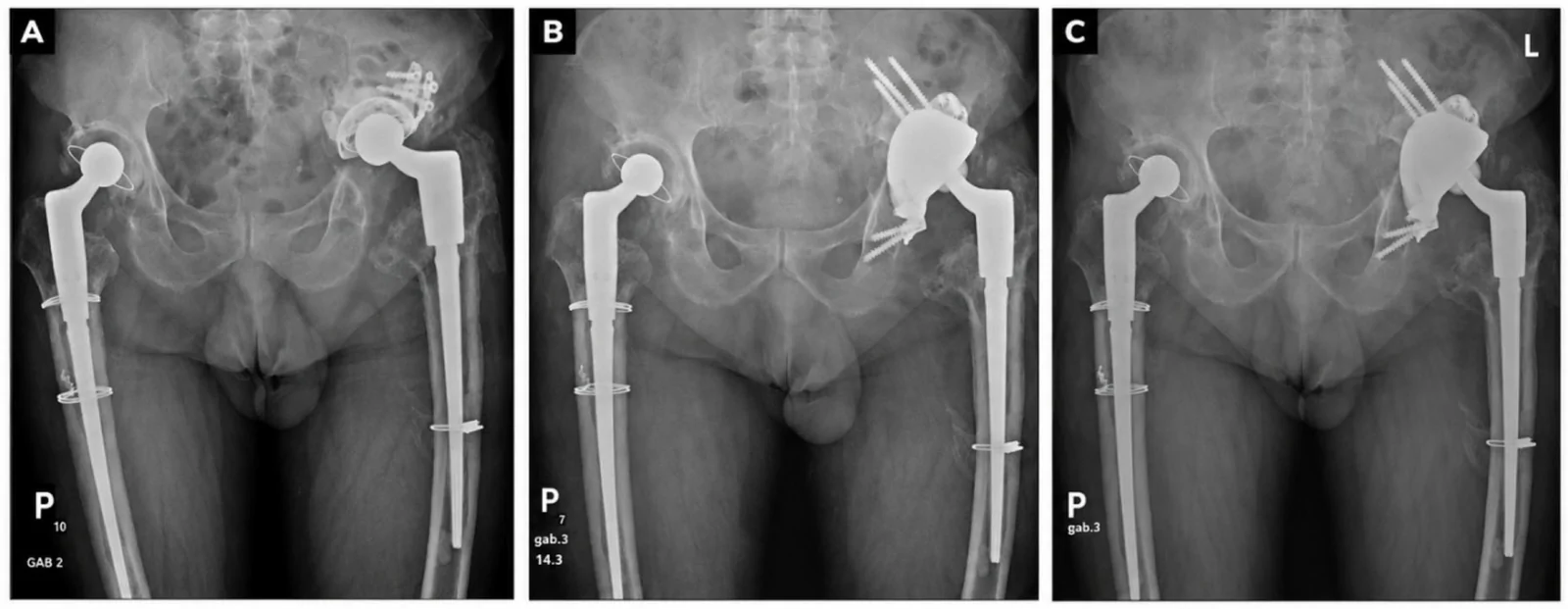
Representative case of a Paprosky type 3B acetabular defect treated with a custom-made 3D-printed implant. (**A**) Preoperative radiograph demonstrating superior migration of acetabular component with extensive bone loss. (**B**) First day after surgery radiograph—3D custom-made reconstruction and (**C**) one year after surgery demonstrating stable implant position and no sign of implant subsidence or loosening. “P” denotes the right side (“prawa” in Polish), and “L” denotes the left side (“lewa” in Polish).

Redon drains were placed in eight patients and were removed once drainage was less than 100 mL/day, usually after 1 to 2 days (mean, 1.8 days) (Table 3, Figure 1 and Figure 2).

**Figure 3:**
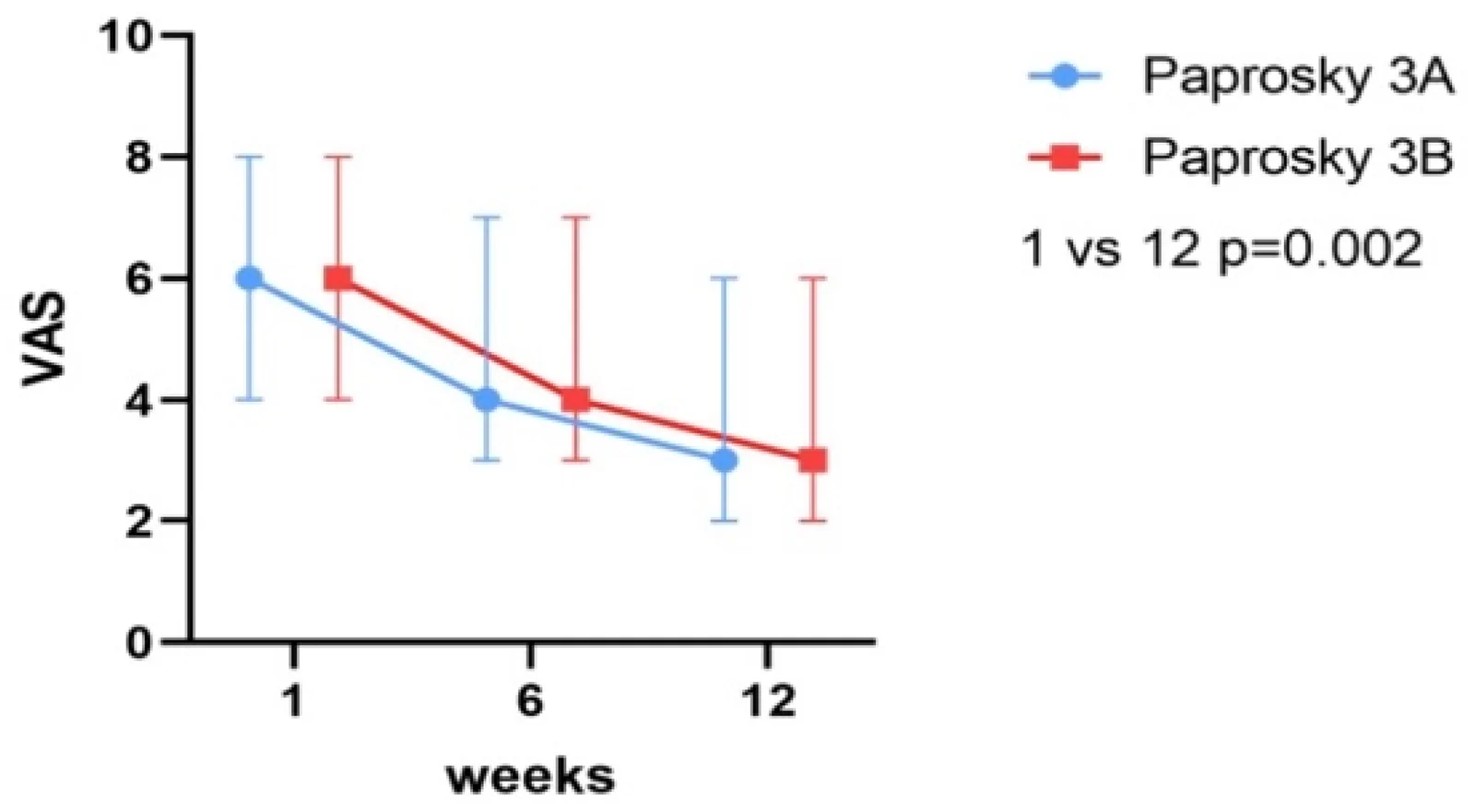
Postoperative assessment of pain in VAS.

**Figure 4:**
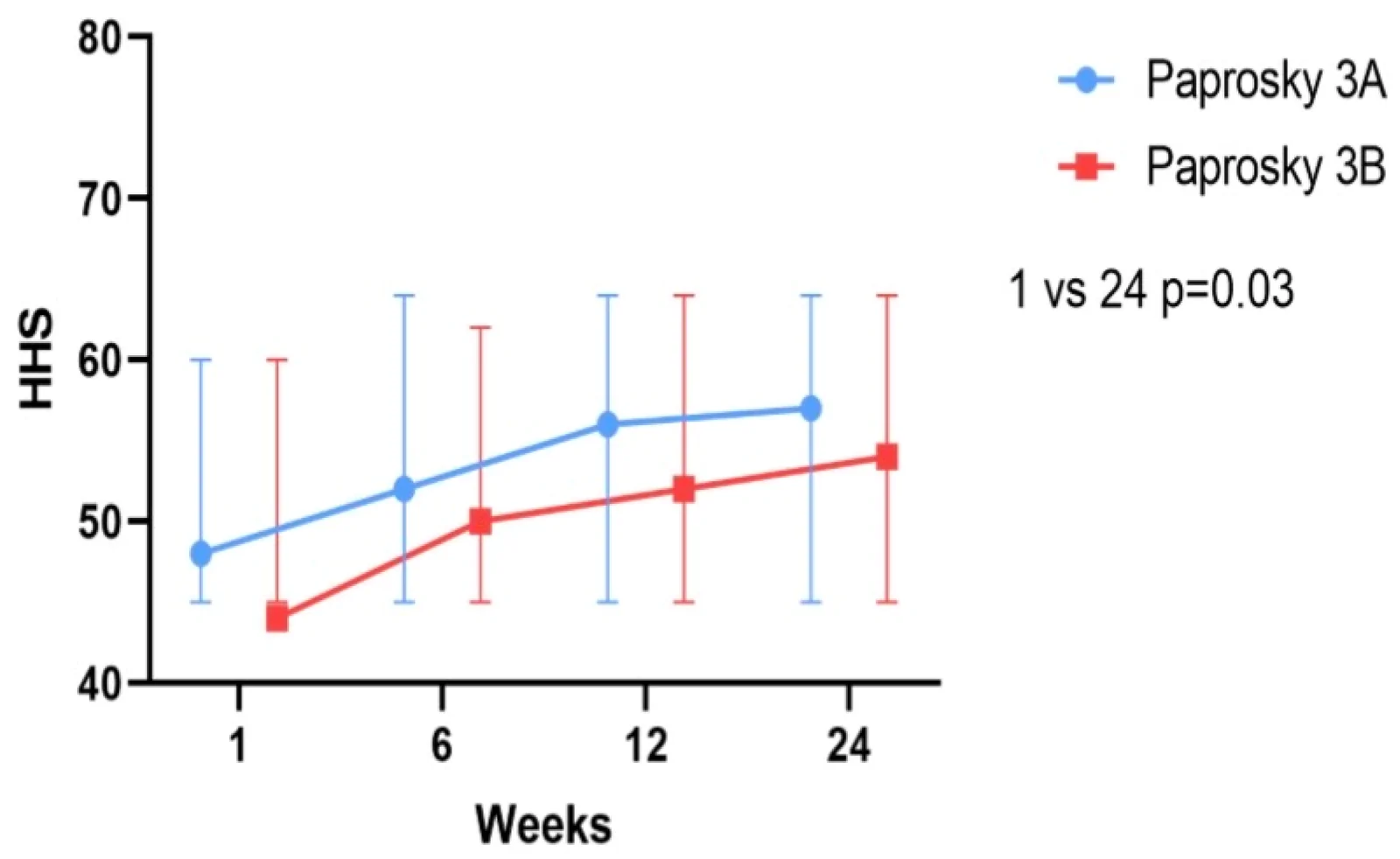
Postoperative assessment of functional status in HHS.

**Figure 5:**
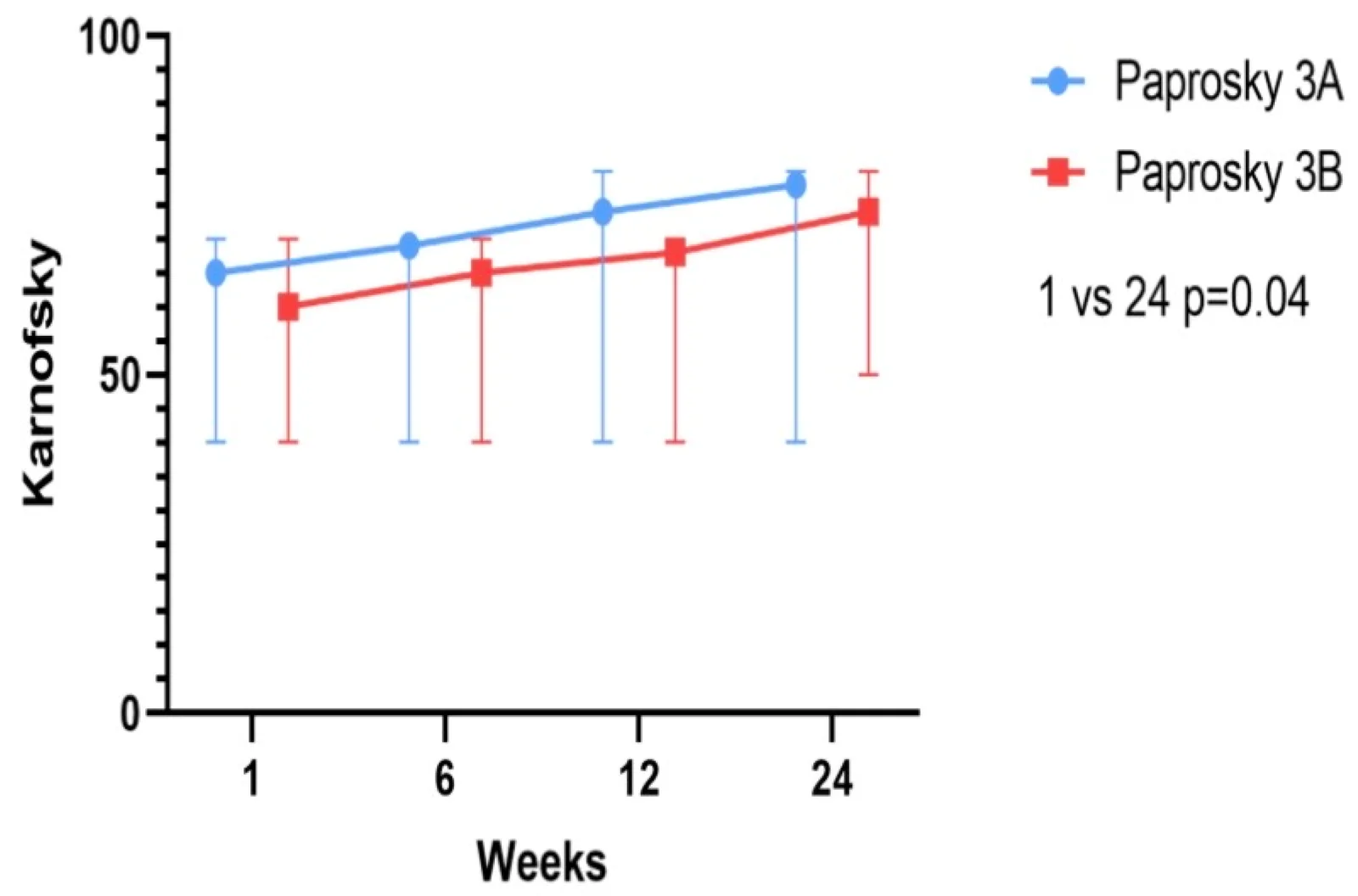
Postoperative assessment of functional status in Karnofsky Score.

A progressive reduction in pain intensity and improvement in mobility were observed throughout follow-up. Functional recovery was accompanied by improvements in HHS and KPS scores, reflecting enhanced mobility, independence, and overall performance status (Figure 3, Figure 4 and Figure 5).

The reported VAS, HHS, and KPS outcomes refer to the standardised assessment period extending to 6 months after surgery.

Radiographic imaging was used to assess prosthesis position and its correspondence with the preoperative plan and the contralateral hip joint. Follow-up assessments were performed at 6 weeks, 3 months, and 6 months after surgery to evaluate implant stability, signs of loosening, and early radiographic findings consistent with progressive implant integration (Table 4).

At 6 months, radial trabecular formation was observed in 26 patients in the Paprosky type 3A group and 21 patients in the type 3B group. These findings were interpreted as early radiographic indicators of implant integration rather than definitive evidence of long-term osseointegration. Postoperative complications were clinically relevant, with a greater numerical burden observed in patients with Paprosky type 3B defects. The most common complications were related to postoperative wound healing and hip dislocation (Table 5).

Wound-healing problems were initially managed with culture-directed antibiotic therapy and negative-pressure wound therapy (NPWT). In the absence of clinical improvement, particularly in patients with persistently elevated inflammatory markers, surgical debridement and exchange of modular prosthetic components were performed as part of a DAIR strategy. Staphylococcus aureus was isolated from cultures in all patients with postoperative wound-healing problems, including those who ultimately required implant removal. Prosthetic dislocations were treated by reduction under general anaesthesia.

## 4. Discussion

Bone defects resulting from aseptic loosening and implant removal are typically irregular and are often covered with granulation tissue and fibrous tissue. Thorough debridement to viable bleeding bone is essential for successful prosthesis integration and may influence long-term outcomes. This is usually performed using curettes and high-speed burrs to prepare the bone bed. In areas of sclerotic bone, drilling may be used to stimulate osteogenesis. In the present study, custom-made 3D-printed acetabular implants provided satisfactory implant positioning and restoration of hip biomechanics in patients with extensive Paprosky type 3A and 3B defects. During the standardised 6-month assessment period, progressive reduction in pain and improvement in functional outcomes were observed, while CT demonstrated early radiographic findings consistent with progressive implant integration. Clinically relevant postoperative complications included wound-healing disorders and prosthetic dislocation, with a greater numerical burden observed in patients with type 3B defects. Because standardised functional and CT assessments were limited to the first 6 months after surgery, these findings should be interpreted as early outcomes and not as definitive evidence of long-term osseointegration or implant survival.

The use of autologous bone grafts in this setting is limited by the often-extensive size of the defects. Alternatively, frozen allografts, bone substitutes, or a combination of both may be used. However, assessment of graft incorporation remains challenging, and their use has been associated with an increased risk of infection [9,10].

Wang et al. identified three key factors influencing successful treatment of acetabular bone defects. The authors emphasised that primary stabilisation of the pelvic ring is the most critical factor, as it enables physiological load transfer and promotes implant osseointegration. In addition, appropriate rehabilitation and early patient mobilisation were considered essential components of successful treatment [21].

Traditional techniques for reconstruction of extensive acetabular bone defects, such as Paprosky type 3A and 3B defects, have been used for many years but are associated with failure rates of up to 37.5% [5]. Commonly used methods include bone grafts, reinforcement cages, multi-hole hemispherical cups, jumbo cups, and titanium augments used in combination with standard acetabular components. Paprosky et al. reported a 4% rate of structural graft loosening in revision of type 3B defects over a mean follow-up of 5.7 years [5]. Lee et al. reported an implant survival rate of 81% for structural graft reconstructions in a cohort of 74 patients, with a mean follow-up of 16 years [22]. Busch et al. reported the outcomes of acetabular reconstruction using cancellous bone grafts and cemented polyethylene cups in a cohort of 42 patients. Implant survival rates were 85% at 20 years and 77% at 25 years [23]. Van Kleunen et al. evaluated the outcomes of treatment using uncemented modular porous metal implants in Paprosky type 2, 3A, and 3B defects over a mean follow-up of 45 months. No cases of aseptic loosening were reported [24]. Landor et al. investigated reconstruction with oblong cups in Paprosky type 3A and 3B defects, with a mean follow-up of 7.3 years. Aseptic loosening was observed in 5.3% to 13.5% of patients, depending on the implant type used [25]. Della Valle et al. reported a 96% implant survival rate over a mean follow-up of 15 years after revision arthroplasty using hemispherical acetabular reconstruction in 131 patients with Paprosky type 2 defects [26]. Löchel et al. reported favourable outcomes in a cohort of 62 patients treated with trabecular metal augments for Paprosky type 2 and 3 acetabular defects. No implant loosening was observed in 92.5% of patients during a 10-year follow-up period [27]. Similar results were reported by Jenkins et al., who demonstrated a 97% cup survival rate at 10 years [28]. The cup-cage technique for acetabular reconstruction was originally described by Sculco et al. During a mean follow-up of 48.8 months, an implant loosening rate of 8% (12 cases) was reported, including eight cases of aseptic loosening [29].

Reconstruction of extensive acetabular bone defects is complex and influenced by multiple factors, including bone loss, impaired vascular supply to the bone and surrounding tissues, and soft tissue damage, particularly involving the musculature. Additional challenges include the need for multiple and extensive surgical approaches, tissue hypovascularity, reduced tissue elasticity, and an increased risk of infection. These procedures are typically lengthy and often require blood transfusion, with patients frequently requiring prolonged hospitalisation. Furthermore, initial implant stability may be insufficient to allow early weight-bearing, which can adversely affect prosthesis integration. Osseointegration may therefore be prolonged, and functional recovery may remain limited for an extended period. In recent years, 3D-printed implants have emerged as a treatment option for extensive acetabular bone defects. These implants enable precise reconstruction of the defect and restoration of the native acetabular geometry. They provide high primary stability, a large bone–implant contact area, and, owing to their porous structure and material properties, facilitate osseointegration. Their high initial stability may allow early full weight-bearing, thereby accelerating functional recovery. Previous studies have highlighted the importance of appropriate load transfer, reduction in micromotion, and optimisation of implant mechanical properties, including stiffness and elasticity, to more closely match those of native bone. Continuous technological advances have led to the development of successive generations of 3D implants, with three generations currently described in the literature [20,30,31]. Taunton et al. reported the use of triflange acetabular implants in a cohort of 57 patients with pelvic discontinuity, achieving an implant survival rate of 95% at a mean follow-up of 76 months [32]. Chiarlone et al. conducted a systematic review including 634 cases and reported that the most common complication was prosthetic dislocation (mean rate, 11.5%), followed by aseptic loosening (2.6%). The overall mean implant survival rate was 94%. The most common indications for implant removal were infection (59%) and aseptic loosening (41%) [33].

The complication profile observed in the present cohort is broadly consistent with previously published reports on custom-made acetabular reconstruction, particularly with regard to prosthetic dislocation and wound- or infection-related complications. However, direct comparison with studies reporting mid- and long-term implant survival should be made cautiously because the standardised radiographic and functional assessment period in the present study was limited to 6 months.

Titanium is the material most commonly used for manufacturing 3D-printed implants. These implants may have a solid structure with porous regions at the bone–implant interface or may be designed with an open lattice structure and a fully porous surface. In some cases, the bone-contact surfaces are additionally coated with hydroxyapatite to further enhance osseointegration. Migaud et al. demonstrated that the rough, porous surface of titanium implants may increase the risk of bacterial colonisation and biofilm formation. In particular, the formation of fluid-filled microenvironments on the implant surface has been observed [34]. In implant design, it is essential to minimise stress at the bone–implant interface resulting from differences in mechanical properties, particularly stiffness and elasticity. Optimisation of this relationship may enhance bone ingrowth and reduce the risk of aseptic loosening. Further advances have been achieved with the introduction of porous fixation elements inserted into bone as an alternative to traditional screw fixation. Another issue requiring further investigation is the potential toxic and carcinogenic effects of implant materials on surrounding tissues [35,36,37,38].

Accurate imaging is essential for successful management of extensive pelvic bone defects. CT is the most commonly used imaging modality, as it enables the creation of three-dimensional pelvic models, implant design, and preoperative planning of the surgical approach. However, artefacts related to previously implanted metal components may impair accurate assessment of bone loss and identification of areas with sufficient bone stock. Precise localisation of regions with adequate bone quality suitable for fixation with screws or pins is critical [39,40,41]. Ivanov et al. used three-dimensional pelvic models for preoperative planning in patients with complex acetabular fractures. This approach facilitated the procedure and reduced the number of complications [42]. Similar results were reported by Zhang et al. in a cohort of 638 patients. Preoperative planning using 3D models enabled a reduction in operative time and more accurate restoration of pelvic anatomy [13]. Wyatt et al. reviewed seven studies including 243 patients who underwent reconstruction of Paprosky type III acetabular defects using 3D-printed implants and reported a low incidence of mechanical failure. The authors emphasised that accurate understanding of pelvic geometry based on three-dimensional modelling is essential for preoperative planning and for achieving favourable clinical outcomes [43]. Myncke et al. reported that the mean duration of procedures involving 3D-printed implants was 300 min, compared with 241 min for standard revision procedures. Prolonged operative time, together with a history of previous infection, was identified as a major risk factor for septic complications [44]. Studies suggest that optimal outcomes are achieved with implants that enable reconstruction of the entire pelvic ring. Secure fixation of the prosthesis to key anatomical structures, such as the femur or sacrum, is essential. In cases involving extensive resection, additional stabilisation of the lumbar spine may be required. Fixation to the ischium and pubis plays a crucial role in overall construct stability, contributing approximately 60% and 40%, respectively. Madanipour et al., in a study with a 30-month follow-up of 46 patients, demonstrated that lack of ischial fixation was associated with increased implant migration and subsequent loosening [40,45].

Preservation of soft tissue structures, particularly muscles, fasciae, and ligaments, plays a crucial role in functional outcomes. It contributes to dynamic prosthetic stability and supports postoperative wound healing. Extensive intraoperative disruption of muscular attachments should be avoided whenever possible. When muscle detachment is unavoidable, reconstruction should be performed using strong sutures anchored to prosthetic components or with suture anchors.

Complications associated with custom-made 3D-printed acetabular reconstruction remain relatively common, although their incidence has decreased over time. The most frequently reported complications include infection, dislocation, postoperative wound-healing disorders, inaccuracies in implant positioning relative to the preoperative plan, insufficient primary stability, and lack of osseointegration.

The use of dual-mobility (DM) or modular dual-mobility (MDM) cups has been shown to reduce the incidence of dislocation, which remains one of the most common complications in this setting. Accurate restoration of the hip COR, as well as appropriate cup inclination and anteversion, is also essential for optimising outcomes. In the present study, dual-mobility articulation was used in all reconstructed hips; nevertheless, prosthetic dislocation remained a clinically relevant complication, indicating that the use of dual-mobility articulation does not completely eliminate the risk of instability in this complex revision population. Wirbel et al. reported 10 cases of deep infection in a cohort of 17 patients treated with custom 3D implants. Dislocation occurred in six patients, and in two cases external hemipelvectomy was required because of severe infection [46]. Bruns et al. reported two cases of nerve palsy in a cohort of 15 patients. One prosthesis was removed because of infection, and two additional infections required open hemipelvectomy [47].

Establishing radiographic criteria for loosening of 3D implants remains challenging. It is important to distinguish implant settling from true loosening. Vertical migration greater than 5 mm, failure of fixation elements (e.g., screws or pins), and fracture of implant flanges are generally considered signs of failed implant integration [35,36,37]. However, the significance of radiolucent lines around fixation elements remains controversial, and their presence alone may not justify revision surgery [38,39]. Careful implant positioning and placement of all planned fixation elements appear to be critical factors in achieving successful osseointegration. Accordingly, the CT findings observed at 3 and 6 months in the present cohort were interpreted as early radiographic indicators of implant integration rather than definitive evidence of mature or long-term osseointegration.

Previous studies have reported a 5-year implant survival rate of approximately 80% [17,31,33,36,41,48,49,50]. Taunton et al. reported a mean HHS of 74.8 points at follow-up beyond 5 years. The authors highlighted the advantages of reconstruction with 3D implants, particularly elimination of prolonged weight-bearing restrictions and reduced reliance on walking aids [32]. Goriainov et al. reported favourable functional and radiographic outcomes following the use of acetabular implants with dual-mobility articulation for the treatment of large periacetabular defects [34]. In a cohort of 19 patients with a mean follow-up of 53 months (range, 17–88 months), a significant improvement in HHS was observed, with a mean postoperative score of 82 points and an implant survival rate of 100%. The authors also suggested that adjunctive use of autologous stem cells may promote de novo bone formation and accelerate implant integration [51].

### Limitations of the Study

The present study has several important limitations. First, its retrospective design is associated with an inherent risk of selection and observational biases. Second, the study group was relatively small, which can be explained by the limited number of patients undergoing such procedures. The small sample size, particularly within individual complication categories, also limits the reliability of comparisons between Paprosky type 3A and 3B defects. Additionally, the absence of a control group limits the interpretation of the findings and precludes direct comparison with alternative approaches. Finally, standardised functional and CT outcome assessments were limited to the first 6 months after surgery. Consequently, definitive conclusions regarding mature osseointegration, long-term implant stability, implant survival, and late complications cannot be drawn. Furthermore, routine preoperative microbiological cultures or biopsies were not obtained; therefore, despite clinical and laboratory screening, occult or low-grade periprosthetic joint infection cannot be excluded with absolute certainty.

## 5. Conclusions

Single-stage reconstruction using custom-made three-dimensional (3D)-printed implants appears to be a feasible treatment option for patients with extensive acetabular bone defects classified as Paprosky type 3A or 3B. In this challenging clinical setting, the technique enabled restoration of acetabular anatomy and hip biomechanics, with early radiographic findings consistent with implant stability and progressive integration, as well as improvement in pain and postoperative mobility. Clinically relevant postoperative complications were observed. Nevertheless, these procedures remain technically demanding and should be undertaken only after careful preoperative planning and in experienced centres. The present findings should be interpreted as early outcomes and do not establish long-term osseointegration or implant survival. Further prospective studies with larger cohorts and longer follow-up are required to determine long-term implant survival, functional outcomes, and complication rates.

## Figures and Tables

**Table 1:** Baseline demographic and clinical characteristics of the study population.

Parameters [Min–Max (Mean)]	Women (*n* = 38)	Men (*n* = 24)	*p*-Value
Age (years)	56–82, (76)	62–84, (74)	0.284
Paprosky 3A [%]	47	67	0.041 *
Paprosky 3B [%]	53	33	0.082
Number of previous surgeries	3–5, (4)	3–8, (4)	0.618
Previous surgical approach	Posterior–16 Anterior–1 Anterior + Posterior–15 Other–0	Posterior–15 Anterior–0 Anterior + Posterior–11 Other–4	
Time since first surgery (years)	9–30, (15)	9–31, (17)	0.356

* *p* < 0.05.

**Table 2:** Preoperative functional status and pain assessment.

Parameter	Paprosky 3A (*n* = 34)	Paprosky 3B (*n* = 28)	*p*-Value
VAS [min–max(mean)]	6–9, (8)	6–9, (8)	0.842
HHS [min–max(mean)]	17–42, (36)	17–38, (34)	0.218
KPS [%]	40–70, (55)	40–70, (53)	0.276
No crutches	0	0	1.000
Onecrutch	4	0	0.041 *
Two crutches	29	25	0.986
Walking frame	1	3	0.038 *
Non-ambulatory	0	0	1.000
Limb shortening [cm]	2–5 (3.2)	2–7 (3.5)	0.327
Muscle insufficiency-gluteal [%]	94	100	0.184

* *p* < 0.05.

**Table 3:** Operative and perioperative parameters.

Parameter [Min–Max (Mean)]	Paprosky 3A (*n* = 34)	Paprosky 3B (*n* = 28)	*p*-Value
Operative time [min]	92–210 (145)	110–260 (175)	0.032 *
Blood loss [mL]	200–1100 (450)	270–2100 (520)	0.044 *
Blood transfusion [%]	30	50	0.048 *
Hospital stay [days]	7–27 (9)	6–24 (9)	0.781
Anaesthesia	Regional–26 General–8	Regional–22 General–6	
Antibiotics	100%	100%	1.000
Tranexamic acid	100%	100%	1.000
Thromboprophylaxis	100%	100%	1.000
Surgical approach	Posterolateral–100%	Posterolateral–100%	1.000
Drainage [N]	3	5	0.472

* *p* < 0.05.

**Table 4:** Postoperative radiographic assessment, restoration of hip biomechanics, and radiographic indicators of implant integration.

Parameter	Paprosky 3A (*n* = 34)	Paprosky 3B (*n* = 28)	*p*-Value
COR shift (vertical) [mm(mean)]	0 + 15 (+2)	−5 + 17, (+3)	0.284
COR shift (horizontal) [mm(mean)]	−5 + 12, (+5)	−5 + 15, (+5)	0.912
Inclination	37–48°, (44)	36–45°, (43)	0.318
Anteversion	0–24°, (16)	4–22°, (14)	0.226
Acetabular offset [mm(mean)]	68–83, (76)	64–78, (74)	0.173
Femoral offset [mm(mean)]	32–48, (46)	34–52, (48)	0.116
Limb length discrepancy [cm(mean)]	−2…+2, (−1)	−3…+2, (−1)	0.874
Radiographic signs of implant loosening (CT)			
Radiolucency > 1 mm (3 months)	12	11	0.731
Subsidence > 5 mm (3 months)	0	3	0.038 *
Screw fracture (3 months)	0	6	0.021 *
Bone fracture (3 months)	0	3	0.038 *
Radiolucency > 1 mm (6 months)	15	18	0.214
Subsidence > 5 mm (6 months)	0	3	0.038 *
Screw fracture (6 months)	0	6	0.021 *
Bone fracture (6 months)	0	3	0.038 *
Radiographic indicators of implant integration (CT)			
Radial trabeculae (3 months)	18	14	0.768
Radial trabeculae (6 months)	26	21	0.834

* *p* < 0.05.

**Table 5:** Early postoperative complications.

Complication [%]	Paprosky 3A (*n* = 34)	Paprosky 3B (*n* = 28)	*p*-Value
Wound healing disorders	6	11	0.041 *
Wound revision	3	6	0.047 *
Prolonged antibiotic therapy	9	18	0.032 *
Infection	6	8	0.044 *
Implant removal	0	4	0.038 *
Dislocation	9	18	0.029 *
Vascular/nerve injury	0	0	1.000
Organ injury	0	0	1.000
Bone fracture	0	4	0.038 *

* *p* < 0.05.

## Data Availability

All data generated or analysed during this study are included in this manuscript.

## Abbreviations

3D	three-dimensional
COR	centre of rotation
CRP	C-reactive protein
CT	computed tomography
DM	dual-mobility
HHS	Harris Hip Score
KPS	Karnofsky Performance Scale
MDM	modular dual-mobility
NSAIDs	non-steroidal anti-inflammatory drugs
THA	total hip arthroplasty
VAS	Visual Analogue Scale
